# T205. CHANGES IN SOCIAL ATTENTION AND EMOTION RECOGNITION FOLLOWING A PILOT SOCIAL SIMULATION COMPUTER GAME INTERVENTION FOR INDIVIDUALS WITH SCHIZOPHRENIA

**DOI:** 10.1093/schbul/sby016.481

**Published:** 2018-04-01

**Authors:** Megan Ichinose, Joshua Wade, Laura Hieber Adery, Lénie Torregrossa, Heathman Nichols, Dayi BIan, Alena Gizdic, Nilanjan Sarkar, Sohee Park

**Affiliations:** Vanderbilt University

## Abstract

**Background:**

Individuals with schizophrenia (SZ) exhibit significant difficulties processing and perceiving socioemotional information conveyed by others. Increasing evidence suggests that SZ deficits in facial emotion recognition, in particular, contribute to impaired daily social functioning. Studies show that improving SZ patients’ visual attention to socially-relevant facial areas (eyes, nose, mouth) with targeted computer interventions will ameliorate deficits in emotion recognition. We tested whether 10 sessions of a novel, VR-based social simulation computer game would indirectly improve facial emotion recognition in SZ, and additionally whether potential gains were associated with changes in gaze patterns.

**Methods:**

Fifteen SZ outpatients completed a social simulation computer game intervention involving a pre-training visit, 10 training sessions scheduled approximately twice per week (days until completion: M=38.8, SD=16), and a post-training visit. During training sessions, participants played a novel, adaptive VR-based computer game that involved approaching and engaging in conversations with various Avatar game characters across several naturalistic settings (a bus stop, café, grocery store). Each game session required completion of 12 “social missions” to determine information about different characters (e.g., food preference), achieved by selecting the appropriate conversational prompts and follow-up questions from multiple options. At pre- and post-training visits, emotion recognition was assessed with a novel dynamic facial affect recognition task (DFAR) and the Bell Lysaker Emotion Recognition Task (BLERT). During the DFAR, participants viewed adult Avatar characters (50% female) making one of 8 dynamic facial expressions (anger, sadness, fear, disgust, joy, surprise, contempt) while gaze data and behavioral responses were recorded. The VR-based computer game and DFAR were developed in-house with Autodesk Maya 3D animation and Unity software (unity3d.com).

**Results:**

Patients’ emotion recognition accuracy (BLERT) significantly improved from pre- to post-training. Patients’ accuracy on the facial affect recognition task (DFAR) also significantly improved following training for specific negative emotions (anger, contempt, fear, and sadness). Regarding changes in visual attention, patients made overall fewer fixations at post-training (fixation duration threshold = 200ms) across relevant social areas (eyes, nose, mouth) when viewing emotional Avatar faces compared to pre-training. A general reduction in fixations was not accompanied by an increase in mean fixation duration. Rather, shorter fixation durations were positively associated with DFAR performance accuracy.

**Discussion:**

SZ patients’ participation in a novel, VR-based computerized social simulation training may yield indirect benefits in emotion recognition. Specifically, patients exhibited improvements on a validated assessment of emotion perception (BLERT) following the 10-session computer training. A decrease in the number of fixations on socially-informative facial regions during Avatars’ emotion expression on the DFAR may indicate an increased efficiency in scanning for socioemotional information. Though much work remains in probing the exact nature of treatment mechanism and durability of these improvements, these promising initial results demonstrate the potential of an VR-based computer game for improving core deficits in social cognition.

